# Rediscovery and reclassification of the dipteran taxon *Nothomicrodon* Wheeler, an exclusive endoparasitoid of gyne ant larvae

**DOI:** 10.1038/srep45530

**Published:** 2017-03-31

**Authors:** Gabriela Pérez-Lachaud, Benoit J. B. Jahyny, Gunilla Ståhls, Graham Rotheray, Jacques H. C. Delabie, Jean-Paul Lachaud

**Affiliations:** 1 El Colegio de la Frontera Sur, Av. Centenario Km 5.5, Chetumal 77014, Quintana Roo, Mexico; 2 Universidade Federal do Vale do São Francisco UNIVASF, Colegiado de Ciências Biológicas, Campus Ciências Agrárias - Rodovia BR 407, 12 Lote 543 Petrolina, Pernambuco, Brazil; 3 Seção de Entomologia, Comissão Executiva do Plano da Lavoura Cacaueira, Centro de Pesquisa do Cacau (CEPLAC, CEPEC), Cx.P.7, 45600-970, Ilhéus, Bahia, Brazil; 4 Finnish Museum of Natural History, Entomology Dept., P.O. Box 17, FIN-00014 University of Helsinki, Finland; 5 National Museums Scotland, West Granton Road, Edinburgh, EH5 1JA, United Kingdom; 6 Laboratório de Mirmecologia, Convênio CEPLAC/UESC, Cocoa Research Center (CEPEC), 45600-000, Itabuna, Bahia, Brazil; 7 Centre de Recherches sur la Cognition Animale, Centre de Biologie Intégrative, Université de Toulouse UPS, CNRS-UMR 5169, 118 Route de Narbonne, 31062 Toulouse Cedex 09, France

## Abstract

The myrmecophile larva of the dipteran taxon *Nothomicrodon* Wheeler is rediscovered, almost a century after its original description and unique report. The systematic position of this dipteran has remained enigmatic due to the absence of reared imagos to confirm indentity. We also failed to rear imagos, but we scrutinized entire nests of the Brazilian arboreal dolichoderine ant *Azteca chartifex* which, combined with morphological and molecular studies, enabled us to establish beyond doubt that *Nothomicrodon* belongs to the Phoridae (Insecta: Diptera), not the Syrphidae where it was first placed, and that the species we studied is an endoparasitoid of the larvae of *A. chartifex*, exclusively attacking sexual female (gyne) larvae. *Northomicrodon* parasitism can exert high fitness costs to a host colony. Our discovery adds one more case to the growing number of phorid taxa known to parasitize ant larvae and suggests that many others remain to be discovered. Our findings and literature review confirm that the Phoridae is the only taxon known that parasitizes both adults and the immature stages of different castes of ants, thus threatening ants on all fronts.

Ants are hosts to at least 17 orders of myrmecophilous arthropods (organisms dependent on ants), ranging from general scavengers to highly selective predators and parasitoids that attack either ants, their brood or other myrmecophiles^1^,^2^,^3^. Recent reviews of ant and myrmecophile relationships reveal both diversity and complexity^4^,^5^,^6^,^7^,^8^,^9^,^10^. The communities inside ant nests and colonies have been likened to homeostatic fortresses or microcosms that encapsulate phenomena normally encountered only at larger scales^1^,^11^. Least studied, however, are ant parasitoid relationships. Compared to other myrmecophiles, few ant parasitoids appear to be entirely successful in evading host colony defense mechanisms^12^,^13^. Unlike other myrmecophiles, ant parasitoids do not integrate with the host colony and have to deal with issues such as locating and successfully parasitizing suitable hosts, and later escaping from the host nest. Some ant parasitoids have mechanisms that are rare in other parasitoids, such as oviposition away from the host combined with a freely mobile, first instar larva (*planidium*) that completes the initial stage of parasitization^12^,^14^. Ant parasitoids can also manipulate host behavior such as provoking in-fighting between worker ants through semiochemicals released by ovipositing females^15^, inducing nest leaving in parasitized workers by developing parasitoids^16^, or reducing host aggressiveness by emerging imagos^17^.

About 750 species, from five taxa of Ecdysozoa (four in Arthropoda and one in Nematoda), are ant parasitoids^18^. Most belong to the Hymenoptera, and a diverse array of families are involved^12^. Dipterans also parasitize ants and representatives of four families are primary parasitoids. Most belong to the Phoridae, for example the so-called ant-decapitating flies of the genera *Apocephalus, Pseudacteon* or *Neodohrniphora* that mostly parasitize workers^19^. Single parasitic species also exist in the Tachinidae (*Strongygaster globula*, an endoparasitoid of colony-founding queens of the genus *Lasius* (Formicinae)^20^), the Syrphidae (*Hypselosyrphus trigonus*, an ectoparasitoid of prepupae of the arboreal ant *Neoponera villosa* (Ponerinae)^21^), and the Chloropidae (*Pseudogaurax paratolmos*, an ectoparasitoid of the larvae of the fungus-growing ant *Apterostigma dentigerum* (Myrmicinae)^22^).

Almost a century ago the enigmatic taxon *Nothomicrodon aztecarum* (Wheeler, 1924) was raised on the basis of morphologically unusual larvae found among the brood of a carton nest ant, *Azteca trigona* (Dolichoderinae), from Barro Colorado Island, Panama^23^. No adult *N. aztecarum* were obtained and due to the similarity in larval stages, Wheeler placed *Nothomicrodon* in the Syrphidae as an ally of the genus *Microdon* whose larvae are well known predators of ant brood^24^. The true affinity of *Nothomicrodon* has remained unresolved because adults have not been reared and larvae have not been re-encountered since its original description. Historically, the genus has been treated as *incertae sedis* by syrphid experts. Cheng & Thompson^25^ stated that the larva has none of the characteristics of microdontine larvae (flattened creeping sole, convex dorsal surface) and based on a suggestion from G.E. Rotheray, speculated that it might belong to the Phoridae. The most up-to-date revision of Microdontinae also treated *Nothomicrodon* as an unplaced taxon^26^.

In this paper we report on the rediscovery of *Nothomicrodon*. Scrutiny of entire nests of *Azteca chartifex* collected in Brazil combined with morphological and molecular studies enabled us to establish beyond doubt that these larvae belong to the Phoridae and that they were endoparasitoids of *A. chartifex* larvae, more specifically of the sexual female (gyne) larvae. Based on these data and a literature review of phorid parasitoids attacking social insect brood, we confirm that the Phoridae is the only insect family known with species that parasitize both the adults and immature stages of their ant hosts, thereby threatening ants on all fronts.

## Results

### DNA sequencing and identification

**Figure 1:**
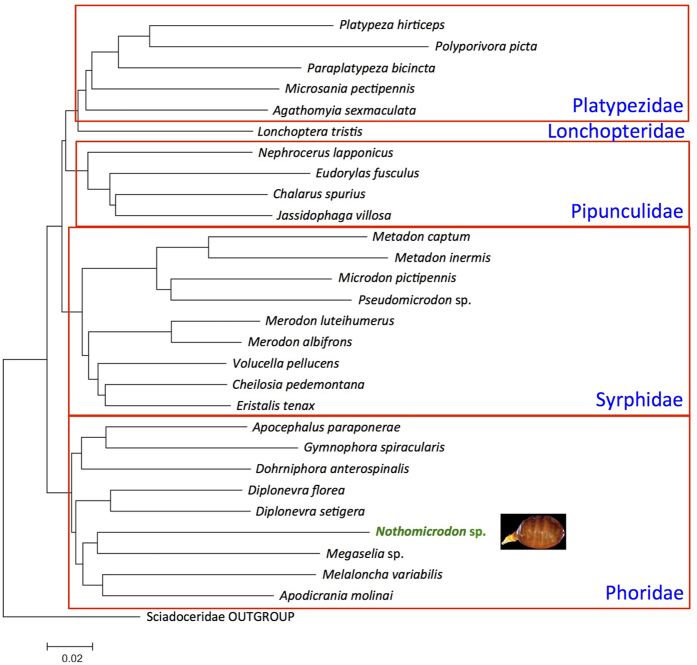
Results of the Neighbor-Joining analysis based on mtDNA COIb sequences. Photo: H. Bahena Basave.

The obtained COIa fragment comprised 653 bp and the COIb 780 bp. The top 20 closest matches of the COIa sequence identification on BOLD were all Phoridae samples (except one Agromyzidae (Diptera: Opomyzoidea) sample). The highest similarity, 88.7%, was found with an unpublished Phoridae sample. Similarities of 88.5% were found with published barcodes of two phorid flies from USA and Canada (BIN BOLD: AAM9347 and BOLD: AAN8679), both from the genus *Megaselia*. Blasting the COIb fragment in GenBank (www.ncbi.nih.gov, on 7 March, 2016) returned a long list of Phoridae samples as closest matches. Sequence similarity of 84% was reported for a sample of the phorids *Anevrina variabilis* (GenBank accession number GU559934) and *Dohrniphora cornuta* (HM352592). Sequence similarity of 83% was reported for two samples of the phorid *Apocephalus paraponerae* (AF217478-9) which is a known ant parasitoid^27^. No syrphid fly species appeared in the top 20 closest matches for both COIa and COIb sequence identification. Moreover, the neighbor-joining and maximum likelihood analyses placed the *Nothomicrodon* sequence among all included samples of other Phoridae (Fig. 1, Table S1), herewith confirming the identification of the sample as a phorid fly, not a syrphid.

### Description of *Nothomicrodon* third instar larva (n = 2)

#### Overall appearance

**Figure 2:**
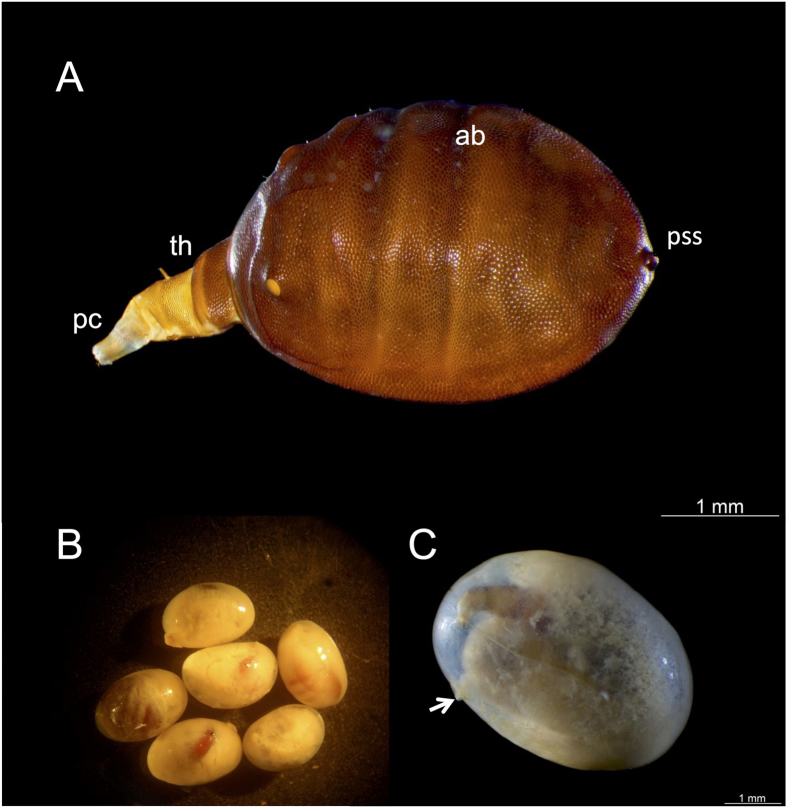
*Nothomicrodon* third instar larva. (**A**) General aspect of whole larva; ab: abdomen; pc: pseudocephalon; pss: posterior spiracular system; th: thorax. (**B**) General aspect of *A. chartifex* gyne larvae parasitized by *Nothomicrodon* (**C**) Fully grown *Nothomicrodon* larva inside an *A. chartifex* larva; arrow points at the host head. Photos: H. Bahena Basave and G. Pérez-Lachaud.

Pear-shaped with a broad, oval-shaped abdomen and a narrow, tapering thorax; pale to brown with a coriaceous integument (Fig. 2A); abdomen smooth except for a single pair of deep infolds across the dorsum (see Fig. S1A); head skeleton with the apex of the labium external to the fleshy pseudocephalon and comprising a pair of downwardly projecting, black, heavily sclerotized hooks, rest of the head skeleton translucent (see Fig. S2A), poorly sclerotized and lacking cibarial ridges.

#### Description

Length about 4.5 mm (1.5 mm pseudocephalon and thorax +3 mm abdomen), abdomen 3.5 mm wide and maximum height about 0.75 mm; width of the metathorax where it joins the broader abdomen, about a quarter the maximum width of the abdomen and at the prothoracic apex, about a fifth the width of the base of the metathorax (see Fig. S1B,C); antennae on the dorso-lateral margins of the pseudocephalon, just posterior to the apex, appearing as a pair of cylindrical, tapering structures (see Fig. S2B), maxillary palpi not recognizable in the specimens examined; ventrally, apex of pseudocephalon with a pair of inwardly directed, flange-like, cuticular projections (see Fig. S1D); pseudocephalon and thorax retractile, as revealed by folds and creases along which the integument probably collapses and/or retracts, by analogy with other larvae^28^; probable margin between the pseudocephalon and prothorax indicated by a deep infold; prothorax elongate, about twice as long as each of the pseudocephalon, mesothorax and metathorax which are all of a similar length (see Fig. S1C); towards the rear of the prothorax on the dorso-lateral margins, are the anterior respiratory processes comprising a pair of cylindrical projections inclined forward and with the spiracles across the apex (see Fig. S1C); metathorax attached to a firm infold at the apex of the first abdominal segment by a band of thin, flexible integument; lateral and posterior margins of the abdomen pinched and with a slight, continuous beading; externally segments marked only by segmental pattern of inconspicuous sensilla, each accompanied by a single hair-like seta, abdomen otherwise unmarked except for the third abdominal segment whose boundaries with adjacent segments are marked by deep infolds across the dorsum (see Fig. S1A); anal segment crescent shaped, as revealed by the pattern of sensilla round the slight, dome-shaped posterior respiratory process; this process with four pairs of short, parallel spiracles orientated dorso-ventrally, above which are a pair of cuticular scars, the paired spiracular plates separated mid-apically by a longitudinal, slit-like infold (see Fig. S2C); entire body coriaceous, locomotory organs apparently absent; head skeleton (see Fig. S2A) 0.5 mm long, form typical for a member of the Platypezoidea^28^; sclerotization slight except for the black, sclerotized apex to the ventral, labial arm which projects externally from the apex of the fleshy pseudocephalon in the form of a pair of stout, downwardly projecting hooks; apex of labrum and mandibles tapered, inconspicuous and insignificant relative to the much larger labial hooks; ventral and dorsal cornua elongate and parallel, not diverging; ventral cornu slightly broader than dorsal cornu; cibarial ridges absent.

#### Taxonomic notes

The third instar larva of our species agrees well with the description and dimensions of the larva of *N. aztecarum*^23^, and we consider it congeneric with this species.

### Life cycle and developmental stages of *Nothomicrodon*

**Figure 3:**
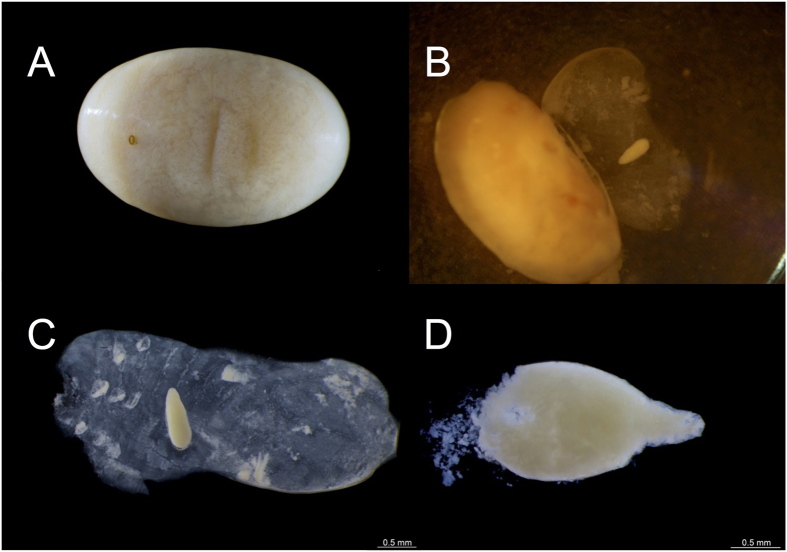
Parasitized *A. chartifex* larva and developmental stages of *Nothomicrodon*. (**A**) *A. chartifex* larva with sclerotized oviposition scar where the posterior respiratory process of the parasitoid fly larva protrudes. (**B**) *Nothomicrodon* egg attached to the host cuticle, the host larva has been dissected. (**C**) Egg. (**D**) First instar larva dissected from its host. Photos: H. Bahena Basave and G. Pérez-Lachaud.

**Figure 4:**
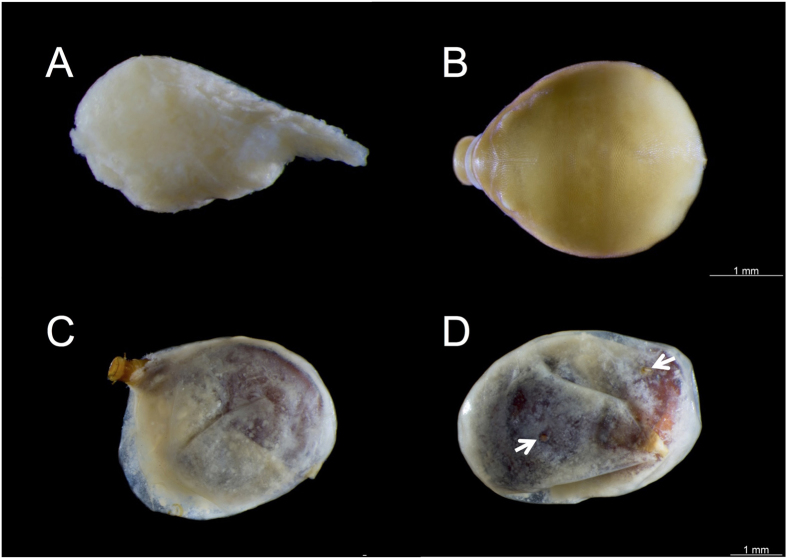
*Nothomicrodon* developmental stages. (**A**) Second instar larva, dissected from the host larva. (**B**) Early third instar larva dissected from the host. (**C**) *Nothomicrodon* third instar larva emerging from the host cuticle remains. (**D**) Two fully grown *Nothomicrodon* larvae inside a single host larva; arrows point at the scars from which the posterior spiracles protrude. Photos: H. Bahena Basave.

Parasitized *A. chartifex* larvae, in both early and advanced stages of parasitoid development, can be identified by the small, oval, melanized/sclerotized scar from which the posterior respiratory process of the parasitoid projects from the host cuticle (Fig. 3A). Advanced stages of development (third instar *Nothomicrodon* larvae) are easily observed through the host integument (Fig. 2B,C). Breathing holes may be located on any part of the ant larva including the head. The hole is round-oval and measures 0.07 mm in diameter on average (n = 7); its rim is raised and heavily sclerotized. Upon host dissection, eggs were found firmly attached to the host cuticle (Fig. 3B,C, n = 2 cases, Table 1). Eggs are elliptical in form with the apical portion more acute than the base. One egg was measured: length = 1.0 mm, base = 0.37 mm and apical portion = 0.19 mm. All developmental stages of *Nothomicrodon* remained attached by the posterior respiratory process to the host cuticle. As with other phorid species^29^, *Nothomicrodon* larva has three instars. The first and second are of a whitish color and the cuticle is not sclerotized (Figs 3D and 4A). First/second instar *Nothomicrodon* larvae dissected from the host measured 1.34 ± 0.09 mm (mean ± SD) in width and 1.88 ± 0.11 mm in length (n = 4). Three of these larvae had the pseudocephalic region extended, length 0.56 ± 0.12 mm. Early third instar larvae are yellow in color (Fig. 4B) and the cuticle has already the leathery aspect of the fully grown, reddish-dark brown third instars (see Fig. 2A). After feeding is completed, third instar larvae cut open the host cuticle with their labial hooks (Fig. 4C). These larvae measure 3.02 ± 0.25 mm in width and 3.51 ± 0.14 mm in length (mean ± SD; n = 8).

### Host caste and developmental stage targeted

**Figure 5:**
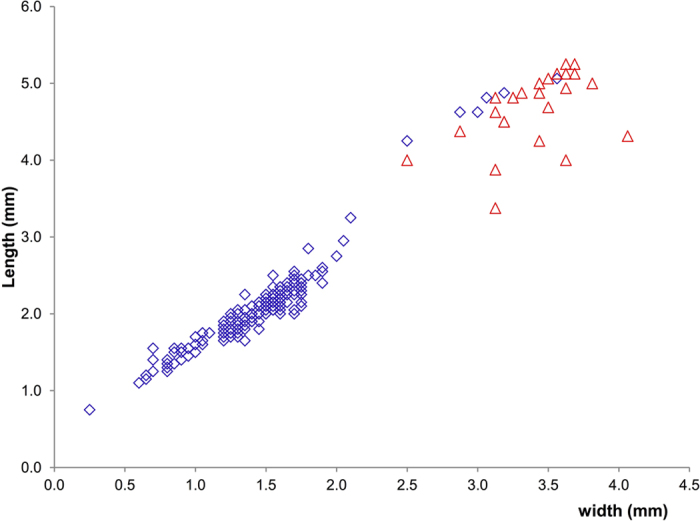
Body length and width of *A. chartifex* larvae. Parasitized larvae in red, unparasitized larvae in blue (n = 212).

*Azteca chartifex* larvae are oval in form and practically hairless; the mouthparts are small, the mandibles are feebly sclerotized and, as in other dolichoderine taxa, mobility is almost lost^30^. Gyne larvae of the Dolichoderinae subfamily are much larger than worker larvae^30^. The length and width of a random sample of larvae of varying sizes were obtained (n = 212). The MDA model discriminated parasitized from unparasitized larvae according to their attributes, with parasitized larvae exclusively in the larger size class, which corresponds to gyne larvae (Fig. 5, see Fig. S3). The model explained 89 and 100% of the between group variance of the variables, and correctly assigned most of the larvae (deviance 19.8, misclassification error 0.94%). Only one parasitized and one unparasitized larvae were not correctly assigned. Parasitized larvae measured on average 3.4 ± 0.3 mm in width and 4.7 ± 0.5 mm in length (mean ± SD; n = 25); unparasitized larvae measured on average 1.5 ± 0.4 mm in width and 2.1 ± 0.6 mm in length (n = 187). *Nothomicrodon* was found only in the nests that contained gyne larvae: no small or fully-grown minor or major worker larvae or male larvae were parasitized.

### Gyne parasitism rates

Samples from three nests collected in 2012 containing a total of 1,328 adults (gynes and workers) and 1,329 larvae and pupae were examined (Table 1). All three samples contained parasitized *A. chartifex* gyne larvae and/or free wandering *Nothomicrodon* larvae (Table 1). In general only one *Nothomicrodon* larva develops per host and in only one occasion two parasitoid larvae were observed inside the same host larva (Fig. 4D). A single *Nothomicrodon* puparium was examined; it was more elliptical in body shape than the larva and seemed to have contracted. This puparium had been parasitized and the parasitoid(s) had emerged and gone as revealed by an emergence hole on its surface (see Fig. S4). Rates of *Nothomicrodon* parasitism for the 2012 samples were calculated as the proportion of parasitized gyne larvae with respect to the total number of examined larvae of this caste (in brackets are the corrected values that take into account wandering *Nothomicrodon* larvae and a puparium). Rates were as follows: sample 1: 54.2% (55.6%), sample 2: 0% (30.8%), sample 3: 100%, with an overall proportion of parasitized gyne larvae of 53.9% (57.9%). Larvae from the 2015 samples were not dissected, and estimated gyne parasitism rates were far lower (Table S2), varying from 3.5 to 75.0% with an overall proportion of parasitized gyne larvae of 8.2% (8.6%).

## Discussion

In this study we resolve the long-standing enigma of the taxonomic placement of Wheeler’s *Nothmicrodon*. Morphological and molecular data reveal that the genus belongs to the Phoridae rather than the Syrphidae where Wheeler^23^ had placed it. Furthermore, our data show that these extraordinary myrmecophilous larvae develop as endoparasitoids of *A. chartifex* larvae, and are specific in only developing on the fully-grown gyne larvae.

The larva we studied shares numerous features with that of *N. aztecarum*^23^ and both molecular and morphological evidence support placement within the Phoridae. For example, the larval head skeleton is of a platypezoid, not a syrphoid form. Specifically, the apex of the head skeleton is the ventral labial arm which is in the form of a pair of large, sclerotized hooks projecting from the pseudocephalon which are the main food gathering structures in platypezoid larvae^28^. The DNA sequences identities and the phylogenetic analyses unambiguously show that the larva belongs to the Phoridae. With a rate of similarity of 83 to 88.5%, our sequences, however, are not closely related to any species represented by mtDNA COI sequences in the public sequence databases, and the adult stage remains to be obtained or assessed.

Species boundaries between members of the host genus *Azteca* are not well understood. *Azteca chartifex* belongs to the *A. trigona* group from which the type material of *Nothomicrodon (N. aztecarum*) was obtained. It remains possible therefore, that the same species of *Nothomicrodon* is associated with both *A. chartifex* and *A. trigona*.

Phorids are a group of small to minute flies comprising ca. 4,300 recognized species but the more conservative estimates consider that this figure may represent only 10% or less of the total fauna when including undescribed species^29^,^31^. They exhibit an array of larval feeding modes, including obligatory and facultative saprophages, predators and parasitoids^29^. Phorids are parasitic on mollusks and arthropod taxa, such as arachnids, millipedes, and insects. They are well known natural enemies of pest ants^32^ and adult honey bees^33^. Most phorid flies associated with ants live either as nest commensals^34^, or as parasitoids of foraging workers^19^ and occasionally alate females^35^. Apart from parasitizing ants, phorids also attack other Aculeata, including bees, stingless bees and wasps^3^. Interestingly, most dipteran parasitoid species attacking social Hymenoptera parasitize the adult stage, although scattered records exist of phorids attacking the larvae of social Hymenoptera (see Tables S3 and S4). About 40% of these cases concern species which develop as ectoparasitoids of formicid and vespid larvae (see Table S3). Larval endoparasitism by phorids is almost exclusively associated with ants (see Table S4). While only two species of the phorid genus *Aenigmatias* (see Table S3 and references therein) are ectoparasitoids of ant larvae, a growing bulk of records now concerns ant larval endoparasitism by phorids (see Table S4 and references therein). The discovery, in this study, of a *Nothomicrodon* species that is an endoparasite of ant larvae hints that other instances of ant larval parasitism exist in phorids. Our results and literature search reveal that the Phoridae is the only family with parasitoid species that attack both adult ants and their broods with, in the case of *Northomicrodon*, a specialization for a specific brood caste, i.e. gyne larvae. Several parasitic wasps (Hymenoptera) also attack adult ants or their brood (larvae or pupae), however, this is achieved by distinct wasp families^12^.

Several morphological features appear to adapt the *Nothomicrodon* larva to a parasitic feeding mode. The labial hooks facilitate piercing, tearing and loosening fragments of host tissue which are then sucked up by the pump in the head skeleton, and guided towards it by the relatively immobile labrum and at either side of it, the tapered mandibles. The long, parallel dorsal and ventral cornua of the head skeleton suggest that it is covered in short, wide muscles. Such a characteristic delivers a shallow but strong pumping action^36^, which is typical of many zoophagous larvae^37^. Perhaps the most distinctive feature of the *Nothomicrodon* larva is its pear shape with an extremely broad, smooth and apparently inflexible abdomen, contrasting with a highly narrowed, tapered, flexible and retractile thorax. Such a shape is also known in larvae of another taxon of cyclorrhaphan endoparasitoids, the Conopidae, whose larvae attack principally the adults of aculeate Hymenoptera^38^. The pear shape in conopid larvae is adaptive in that the broad abdomen resides in the abdomen of the host while the narrow thorax reaches through the petiole into the thorax to feed on the high density of muscle tissue. The pear shape of the *Nothomicrodon* larva appears to be similarly adapted. Flexibility in the thorax probably facilitates reaching in and around the host body in order to gather food, and might also help egression from the host after completion of larval growth. The *Nothomicrodon* larva might well eat the host remains surrounding its body, as occurs in other parasitoids, such as the braconid wasp *Toxoneuron bicolor* (=*Toxoneuron nigriceps*). In this endoparasitoid, post-egression feeding enhances growth and survival^39^. However, on the basis of mouthpart structure, it would likely be difficult for the *Northomicrodon* larva to fragment the host remains, unlike the braconid which has chewing mandibles. In the *Nothomicrodon* larva, the absence of locomotory structures on the ventrum of the abdomen is possibly explained by the position of the larva inside a host where locomotion is not required. The relative size of the anterior respiratory processes is surprising given the position of the thorax inside the host. In contrast, the posterior respiratory process projects only slightly which is probably an adaptive shape in that it is less likely to become caught in host tissue. The flat ventral and slightly convex dorsal surfaces of the larva, together with their hard, leather-like cuticle, and the possibility of retraction of the pseudocephalon and thorax (the most fragile parts of the body) seem to be adaptations to live on the very hard and concave carton walls of the host nest chambers, and might well provide protection from aggressive worker ants.

Whether the *Nothomicrodon* female places its eggs near the host (or host habitat) and the fly larva actively seeks for its host, or lays eggs directly on an *Azteca* ant larva within the host nest remains to be assessed. Whatever, our results show that only the fully-grown gyne larvae of the ant host are targeted, and suggest that host size may be a limiting factor to *Nothomicrodon* larval development. Ant parasitoids impose variable fitness costs on both individuals and colonies^13^,^33^,^40^,^41^. For high rates of parasitism, parasitoids may significantly reduce resource intake, colony size, and colony fitness. By exclusively parasitizing gyne larvae, *Nothomicrodon* parasitism directly affects the reproductive success of the colony and thereby imposes a high fitness cost to *A. chartife*x. Other parasites and parasitoids impose high reproductive costs on their hosts as for example, in *Nosema* infections of bumble bees^42^. However, fitness costs are not inevitable; not all *A. chartifex* colonies we studied suffered high rates of *Nothomicrodon* parasitism.

## Materials and Methods

### Insect sampling and preparation

*Azteca chartifex* adults and brood, as well as *Nothomicrodon* larvae, were collected in the state of Bahia in Brazil in 2012 and 2015 (S*I Text: Material and Methods*). *Azteca* workers, larvae and pupae were examined under a stereomicroscope (Nikon SMZ745T) and dissected if parasitized (Table 1). Late instar *Nothomicrodon* larvae found in the nest galleries along with workers and ant brood were collected and examined. Ant larvae collected in 2015 were checked only externally, without dissection, and used essentially for estimating gyne parasitism rates (see Table S4). *Nothomicrodon* larvae and a subsample of *A. chartifex* larvae (including both parasitized and unparasitized larvae) were measured to the nearest 0.1 mm (width × length) using a stereomicroscope provided with an ocular micrometer. A Mixture Discriminant Analysis (MDA) model was fitted to the *A. azteca* data set to test for differences in size between parasitized and unparasitized larvae. MDA is effective for selecting the suitable subclass division of a data set (Gaussian mixture of subclasses)^43^. The statistical analysis was performed using the MDA package in R^44^. An overall gyne parasitism rate was calculated taking into account all of the potential host larvae examined, with a correction for parasitoid larvae/pupae found freely in the nest chambers. *Nothomicrodon* larvae preserved in alcohol were prepared for description by rehydration and then fixation in Kahle’s solution^38^. To examine the larval head skeleton, the apex of the thorax of a preserved larva was cut off and soaked in hot KOH for about 5 minutes. Excess tissue was removed from the head skeleton and it was washed in acetic acid and stored in glycerol. It was examined using a Wild 5 stereo-microscope in a solid watch glass containing 70% ethanol. The drawing was made with a drawing tube attached to the microscope. Terminology generally follows Rotheray & Gilbert^28^. Stacked images were obtained using Helicon Focus© (Helicon Soft Ltd). Specimens were also critical point dried and sputter coated before observation with a SM-51 TOPCON Scanning Electron Microscope.

### DNA sequencing, identification and clustering

Three second instar larvae of *Nothomicrodon*, obtained by dissection of the hosts, were used for molecular work (labelled Aztc 017B-I, Aztc 017B-II and Aztc 017B-III). DNA was extracted from a small piece of tissue (0.5–1.0 mm sample) of the larvae using the Phire™ Tissue Direct PCR master Mix #F-170S kit (Thermo Scientific Baltics UAB, Vilnius, Lithuania) following the Dilution & Storage protocol with some modifications (*SI Text: Material and Methods*).

The obtained COIa and COIb sequence fragments of our species of *Nothomicrodon*, referred as *incertae sedis* in Table S1, were individually blasted against the BOLD systems v3 (boldsystems.org, accessed 7 March, 2016) and the NCBI GenBank databases, respectively, using BLASTn for the sequence comparisons and identifications. Sequences produced in this study were deposited in the European Nucleotide Archive (http://www.ebi.ac.uk/ena), accession numbers LT592267 (COIa) and LT592268 (COIb).

We additionally used a dataset of COIb sequences retrieved from GenBank with the aim to test the placement of our species of *Nothomicrodon* among samples of the closely related cyclorrhaphan fly families using sequence clustering. The dataset comprised eight COIb sequences of Phoridae species, nine of Syrphidae, five of Platypezidae, four of Pipunculidae, one of Lonchopteridae, and used Sciadoceridae as outgroup (Table S1). The COIb sequence dataset comprised 764 nucleotides and was analyzed using Neighbor-Joining and Maximum Likelihood in software MEGA v.6^45^ using the K2P and General Time Reversible^46^ models of evolution, respectively.

Third instar larvae of our species were further compared to the description and figures of *N. aztecarum* in Wheeler^23^ and to the images of the paratype in the Syrphidae Community Website http://syrphidae.myspecies.info/taxonomy/term/75. Voucher material of ants and parasitoids was deposited in the following collections: Centro de Pesquisa do Cacau at Ilhéus, Brazil (CPDC collection, CEPEC/CEPLAC) (10 host workers, five third instar *Nothomicrodon* larvae); El Colegio de la Frontera Sur at Chetumal, Mexico (Colección de Formicidae and Colección de Artrópodos) (10 host workers, three third instar *Nothomicrodon* larvae); the National Museums at Edinbugh, Scotland (three third and one second instar *Nothomicrodon* larvae); and the Finnish Museum of Natural History at Helsinki (Finland) (three second instar *Nothomicrodon* larvae, 2 host workers).

## Additional Information

**How to cite this article:** Pérez-Lachaud, G. *et al*. Rediscovery and reclassification of the dipteran taxon *Nothomicrodon* Wheeler, an exclusive endoparasitoid of gyne ant larvae. *Sci. Rep.* **7**, 45530; doi: 10.1038/srep45530 (2017).

**Publisher's note:** Springer Nature remains neutral with regard to jurisdictional claims in published maps and institutional affiliations.

## Supplementary Material

Supplementary Information

## Figures and Tables

**Table 1:** *Azteca chartifex* material examined for this study, gyne parasitism rate and number and developmental stage of *Nothomicrodon*.

Nest ident.	Gynes	Gyne pupae	Workers	Worker larvae	Gyne larvae	Total larvae	Parasitized gyne larvae	Gyne parasitism rate (%)	Corrected gyne parasitism rate (%)^a^	Developmental stage of *Nothomicrodon*	*Nothomicrodon* L_3_ wandering in the host nest	
Aztc 017	0	0	1295	1170	96	1266	52	54.2	55.6	2 eggs, 13 L_1-2_, 12 early L_3_, 25 L_3_	3	
Aztc 032	0	0	0	0	9	9	0	0	30.8		3	1 parasitized puparium
Aztc 033	7	44	26	0	10	10	10	100	100	10 L_3_	4	
Total	7	44	1321	1170	115	1285	62	53.9	57.9	62	10	1

^a^Corrected to take into account the free wandering *Nothomicrodon* larvae and a puparium.
