# Supplementary material for: Rediscovery and reclassification of the dipteran taxon Nothomicrodon Wheeler, an exclusive endoparasitoid of gyne ant larvae
Source: Sci Rep. 2017 Mar 31;7:45530. doi: 10.1038/srep45530 (PMC5374537; doi:10.1038/srep45530)
Supplement: Supplementary Information [file srep45530-s1.pdf]

Supplementary Information

Rediscovery and reclassification of the dipteran taxon *Nothomicrodon* Wheeler, an exclusive endoparasitoid of gyne ant larvae

Gabriela Pérez-Lachaud[1], Benoit J. B. Jahyny[2,3], Gunilla Ståhls[4], Graham Rotheray[5], Jacques H. C. Delabie[6] & Jean-Paul Lachaud[1,7]

[1] El Colegio de la Frontera Sur, Av. Centenario Km 5.5, Chetumal 77014, Quintana Roo, Mexico

[2] Universidade Federal do Vale do São Francisco UNIVASF, Colegiado de Ciências Biológicas, Campus Ciências Agrárias - Rodovia BR 407, 12 Lote 543 Petrolina, Pernambuco, Brazil

[3] Seção de Entomologia, Comissão Executiva do Plano da Lavoura Cacaueira, Centro de Pesquisa do Cacau (CEPLAC, CEPEC), Cx.P.7, 45600-970, Ilhéus, Bahia, Brazil

[4] Finnish Museum of Natural History, Entomology Dept., P.O. Box 17, FIN-00014 University of Helsinki, Finland

[5] National Museums Scotland, West Granton Road, Edinburgh, EH5 1JA, United Kingdom

[6] Laboratório de Mirmecologia, Convênio CEPLAC/UESC, Cocoa Research Center (CEPEC), 45600-000, Itabuna, Bahia, Brazil

[7] Centre de Recherches sur la Cognition Animale, Centre de Biologie Intégrative, Université de Toulouse UPS, CNRS-UMR 5169, 118 Route de Narbonne, 31062 Toulouse Cedex 09, France

Content

SI Text. Materials and Methods.

Figure S1. SEM micrographs of a *Nothomicrodon* third instar larva.

Figure S2. Details of a *Nothomicrodon* third instar larva.

Figure S3. Two-dimensional view of the MDA model fitted to body size measurements.

Figure S4. *Nothomicrodon* puparium.

Table S1. List of included taxa and GenBank accession numbers for COIb sequences,

Table S2. *Azteca chartifex* complementary material.

Table S3. Phorid ectoparasitoids of social insects larvae,

Table S4. Phorid endoparasitoids of social insects larvae,

Table S5. *Azteca chartifex* nests that contained *Nothomicrodon*.

SI Text

Materials and Methods

Ethics Statement

Collection and transport of insect specimens involved in this research were authorized by the Brazilian Chico Mendes Institute for Biodiversity Conservation (SISBIO/ICMBio Permit Number 11304-1 to JHCD). The collection did not involve endangered or protected species. Research and field work comply with the current laws of Brazil.

Azteca chartifex *natural history*

The dolichoderine genus *Azteca* is an exclusively Neotropical group of arboreal ants, distributed from Mexico to Argentina[1]. They are omnivorous, but have a preference for

glucid-rich liquid diets[2]. The biology of the medium sized *A. chartifex* is not well understood yet. This territorial species belongs to the *A. trigona* group, for which various myrmecophilous associates have been reported[3]. It is common in wet forests of northern South America, from Costa Rica to southern Brazil[1], and dominant in shaded cocoa plantations in Brazil[4]. Colonies are polydomous and occur in clusters of large, conspicuous, pendant carton nests that frequently assume a conical form[5,6]. The largest unit can extend more than 2 meters in height[7] and house several thousands of workers. Two groups of workers are clearly distinguished (minors and majors) and physical differences in size are reflected in a clear division of labor, with foragers composed mainly by majors (80%)[7]. Major workers body length is 3.07 ± 0.07 mm[8]. A unique physogastric queen is found in a large chamber near the attachment point on the supporting tree branch; however the queen was observed in only a few nests (B.J.B.J. and J.H.C.D. unpubl. data).

Insect sampling and preparation

Azteca chartifex adults and brood, as well as *Nothomicrodon* larvae, were gathered during a large scale study on ant biodiversity at the Cocoa Research Center (CEPEC-CEPLAC) field station, Ilhéus, Bahia, Brazil. From mid February to October 2012, two *Azteca* nests were collected each month (n = 17). Additionally, at the end of October and early November 2015, nine more nests were harvested for complementary data. Nests were revised in the laboratory in search for myrmecophilous organisms. More specifically, material containing *Nothomicrodon* examined for this study comprised a subset of larvae and adult ants from three nests collected in 2012 and six nests collected in 2015 (see Table S5 for details).

DNA sequencing and identification

The Phire™ Tissue Direct PCR master Mix is designed to perform PCR directly from tissue samples with no prior DNA purification. The tissue sample was placed in an Eppendorf tube in 30 µl of Dilution Buffer, and 0.8 µl of DNA Release Additive was added. The tube was briefly vortexed and centrifuged, incubated at room temperature for about 20 min and then placed in a pre-heated block at 98°C for 2 min, and finally centrifuged at 11 000 rpm for 1 min; 1 µl of supernatant was used in a 20 µl PCR reaction. The cycling conditions were initial denaturation at 98°C for 5 min, 40 cycles of denaturation at 98°C for 5 s, annealing at 49°C for 30 s, extension at 72°C for 20 s, and final extension at 72°C for 1 min. The Folmer fragment or "barcode fragment" of the 5' region of COI (= COIa) was amplified with forward primer LCO1490 (5'-GCTCAACAAATCATAAAGATATTGG-3') and reverse primer HCO2198 (5'-TAAACTTCAGGGTGACCAAAAAATCA-3')[9], and the COIb fragment was amplified using forward primer C1-J-2183 (5'-CAA CAT TTA TTT TGA TTT TTT GG-3') (alias JERRY) and the reverse primer TL2-N-3014 (5'-TCC AAT GCA CTA ATC TGC CAT ATT A-3') (alias PAT)[10]. Amplified PCR products were electrophoresed on 1.5% agarose gels and treated with Exo-SapIT (USB Affymetrix, Ohio, USA) prior to sequencing. Both PCR primers were used for sequencing. The Big Dye Terminator Cycle Sequencing Kit (version 3.1) (Applied Biosystems, Foster City, CA, USA) was used on an ABI 3730 (Applied Biosystems, Foster City, CA, USA) genetic analyzer at the Sequencing Service Laboratory of the Finnish Institute for Molecular Medicine (www.fimm.fi). The sequences were edited for base-calling errors and assembled using Sequencher™ (version 5.0) (Gene Codes Corporation, Ann Arbor, MI, USA).

1. Longino, J. T. A taxonomic review of the genus *Azteca* (Hymenoptera: Formicidae) in Costa Rica and a global revision of the *aurita* group. *Zootaxa* **1491**, 1–63 (2007).

2. Harada, A. Y., Benson, W. W. Espécies de *Azteca* (Hymenoptera: Formicidae) especializadas em *Cecropia* spp. (Moraceae): distribuição geográfica e considerações ecológicas. *Rev. Bras. Entomol.* **32**, 423–435 (1988).
3. Wheeler, W. M. Two extraordinary larval myrmecophiles from Panama. *Proc. Natl Acad. Sci. USA* **10**, 237–244 (1924).
4. Majer, J. D., Delabie, J. H. C. & Smith, M. R. B. Arboreal ant community patterns in Brazilian cocoa farms. *Biotropica* **26**, 73–83 (1994).
5. Delabie, J. H. C., Benton, F. P. & Medeiros, M. A. La polydomie chez les Formicidae arboricoles dans les cacaoyères du Brésil: optimisation de l'occupation de l'espace ou stratégie défensive? *Actes Coll. Ins. Soc.* **7**, 173–178 (1991).
6. Delabie, J. H. C. *et al.* Contribution of cocoa plantations to the conservation of native ants (Insecta: Hymenoptera: Formicidae) with a special emphasis on the Atlantic Forest fauna of southern Bahia, Brazil. *Biodivers. Conserv.* **16**, 2359–2384 (2007).
7. Wheeler, D. E. Polymorphism and division of labor in *Azteca chartifex laticeps* (Hymenoptera: Formicidae). *J. Kansas Entomol. Soc.* **59**, 542–548 (1986).
8. Vencl, F. V., Nogueira-de-Sá, F., Allen, B. J., Windsor, D. M. & Futuyma, D. J. Dietary specialization influences the efficacy of larval tortoise beetle shield defenses. *Oecologia* **145**, 404–414 (2005).
9. Folmer, O., Black, M., Hoeh, W., Lutz, R. & Vrijenhoek, R. DNA primers for amplification of mitochondrial cytochrome *c* oxidase subunit I from diverse metazoan invertebrates. *Mol. Mar. Biol. Biotech.* **3**, 294–299 (1994).
10. Simon, C. *et al.* Evolution, weighting, and phylogenetic utility of mitochondrial gene sequences and a compilation of conserved polymerase chain reaction primers. *Ann. Entomol. Soc. Am.* **87**, 651–701 (1994).

11. Enderlein, G. *Oniscomyia dorni*, eine neue deutsche als Ameisengast lebende flügellose Fliegengattung, sowie über die systematische Stellung der *Thaumatoxena*. *Zool. Jahrb.* **27**, 145–156 (1908).

12. Wasmann, E. Weitere Beiträge zum sozialen Parasitismus und der Sklaverei bei den Ameisen. *Biol. Centralbl.* **28**, 726–731 (1908).

13. Donisthorpe, H. *Aenigmatias blattoides* Meinert, captured in Scotland. *Entomol. Rec.* **25**, 277–278 (1913).

14. Donisthorpe, H. Some notes on the genera *Platyphora*, Verrall, and *Aenigmatias*, Meinert, and a species new to Britain. *Entomol. Rec.* 26, 276–278 (1914).

15. Donisthorpe, H. St J. K. *The Guests of British Ants. Their habits and life histories*. (G. Routledge & Sons, London, UK, 1927).

16. Falk, S. J. & Chandler, P. J. A review of the scarce and threatened flies of Great Britain. Part 2: Nematocera and Aschiza not dealt with by Falk (1991). *Species Status* **2**, 1–189 (2005).

17. Schmitz, H. Die myrmecophilen Phoriden der Wasmann'schen Sammlung. *Zool. Jahrb.* **37**, 509–566 (1914).

18. Nelson, J. M. Parasites and symbionts of nests of *Polistes* wasps. *Ann. Entomol. Soc. Am.* **61**, 1528–1539 (1968).

19. Richards, O. W. & Richards, M. J. Observations on the social wasps of South America. *Trans. R. Ent. Soc. Lond.* **102**, 1–169 (1951).

20. Jeanne, R. L. Descriptions of the nests of *Pseudochartergus fascatus* and *Stelopolybia testacea* with a note on a parasite of *S. testacea* (Hymenoptera. Vespidae). *Psyche* **77**, 54–69 (1970).

21. Little, M. Social biology of the polistine wasp Mischocyttarus labiatus: survival in a Colombian rain forest. *Smith. Contr. Zool.* **327**, 1–27 (1981).

22. Williams, R. N. Insect natural enemies of fire ants in South America with several new records. *Proc. Tall Timbers Conf. Ecol. Anim. Contr. Habit. Manage.* **7**, 123–134 (1980).

23. Williams, R. N. & Whitcomb, W. H. Parasites of fire ants in South America. *Proc. Tall Timbers Conf. Ecol. Anim. Contr. Habit. Manage.* **5**, 49–59 (1974).

24. Packard, A. S., Jr. The parasites of the hobey-bee. *Am. Nat.* **2**, 195–205 (1868).

25. Thompson, F. Some notes on the hunchback fly (*Phora incrassata*, Mg.). *Bee World* **19**, 19–23 (1938).

26. Wheeler, G. C. & Wheeler, J. The ant larvae of the subfamily Formicinae. *Ann. Entomol. Soc. Am.* **46**, 126–171 (1953).

27. Lachaud, J.-P. & Pérez-Lachaud, G. Ectaheteromorph ants also host highly diverse parasitic communities: a review of parasitoids of the Neotropical genus *Ectatomma*. *Insect. Soc.* **62**, 121–132 (2015).

28. Wheeler, G. C. & Wheeler, J. The ant larvae of the subfamily Ponerinae — Part I. *Am. Midl. Nat.* **48**, 111–144 (1952).

29. Wheeler, G. C. & Wheeler, J. The ant larvae of the subfamily Dolichoderinae: Supplement. *Ann. Entomol. Soc. Am.* **59**, 726–732 (1966).

30. Wheeler, G. C. & Wheeler, J. Ant larvae: review and synthesis. *Mem. Ent. Soc. Wash.* **7**, 1–108 (1976).

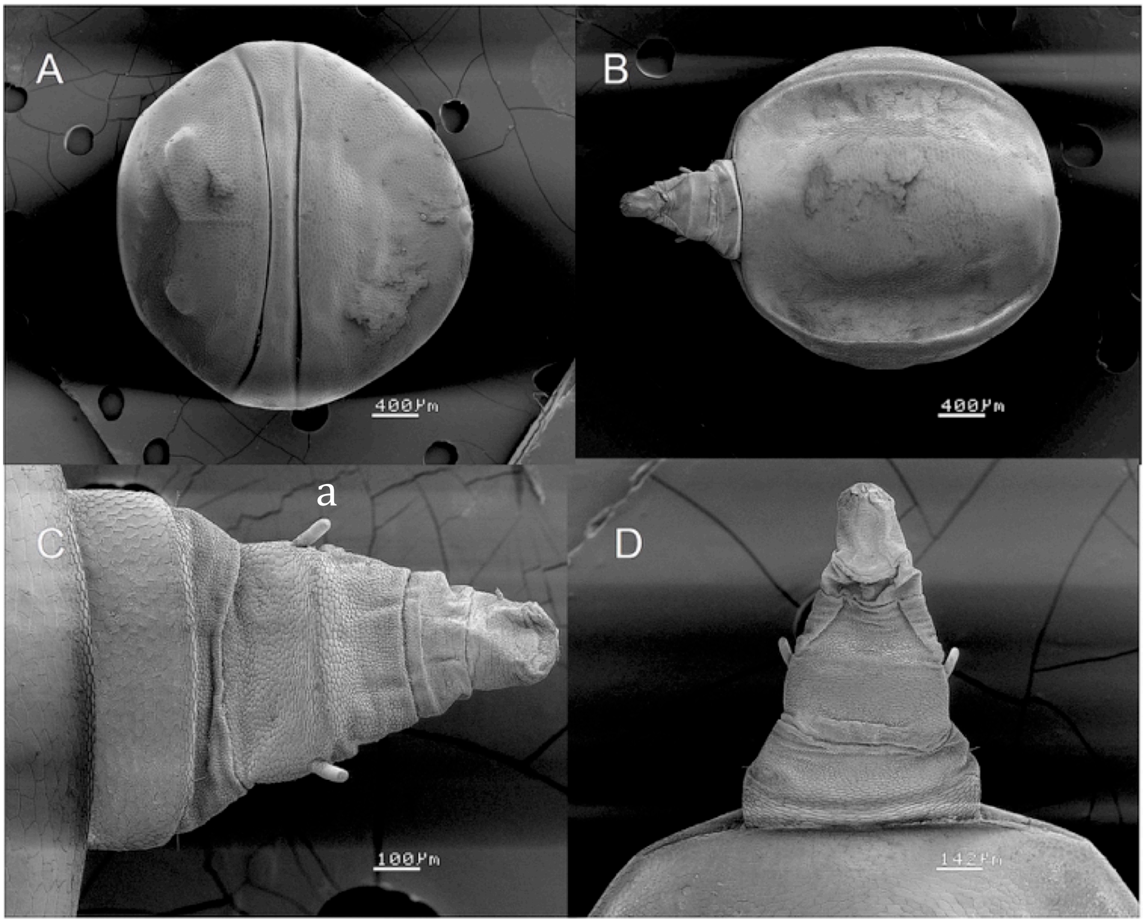

Figure S1. SEM micrographs of a *Nothomicrodon* third instar larva. (A) Whole larva, dorsal view (pseudocephalon and thorax retracted). (B) Whole larva, ventral view. (C) Pseudocephalon and thorax, dorsal view; a: anterior respiratory process. (D) Pseudocephalon and thorax, ventral view. Photos: Guadalupe Nieto.

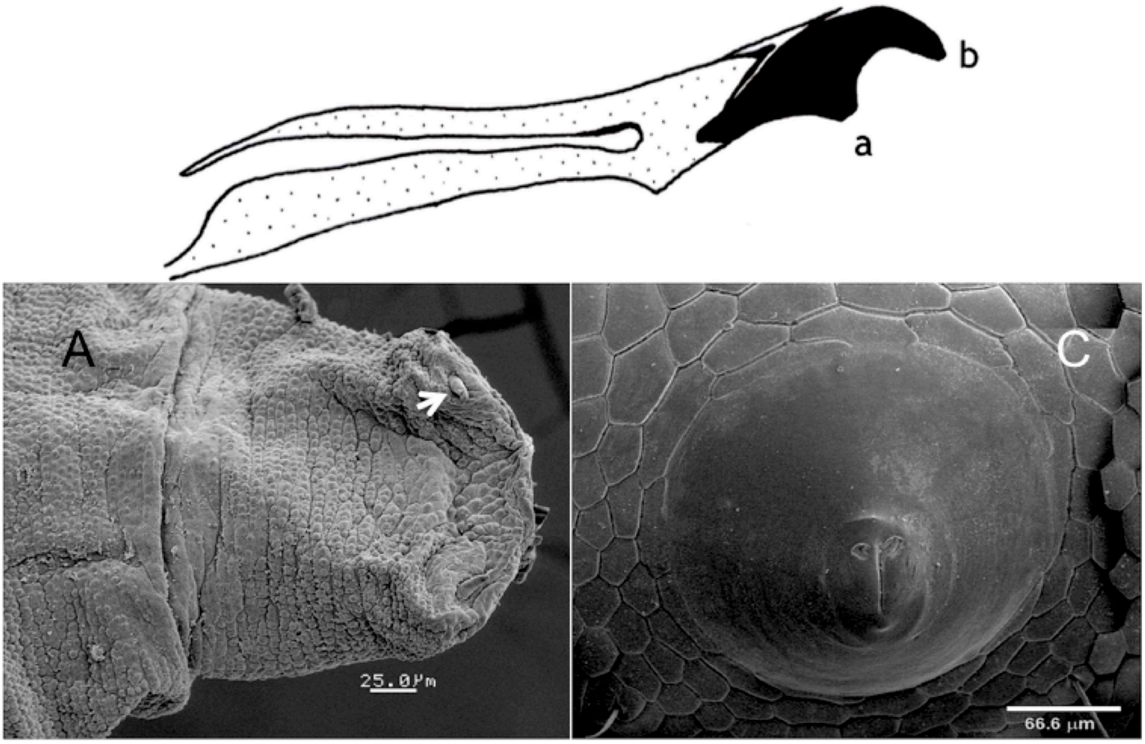

Figure S2. Details of a *Nothomicrodon* third instar larva. (A) Drawing of lateral view of the head skeleton, actual length 0.5 mm. The black hooks are not mandibles, they are the sclerotized apex of the ventral labial arm, a platypezoid feature supporting the molecular work showing that this is a phorid; a: ventral labial arm; b: labial hook. (B) SEM micrograph of pseudoce-phalon, dorsal view; the arrow points at the left antenna. (C) SEM micrograph of the posterior spiracular system. Photos: Guadalupe Nieto.

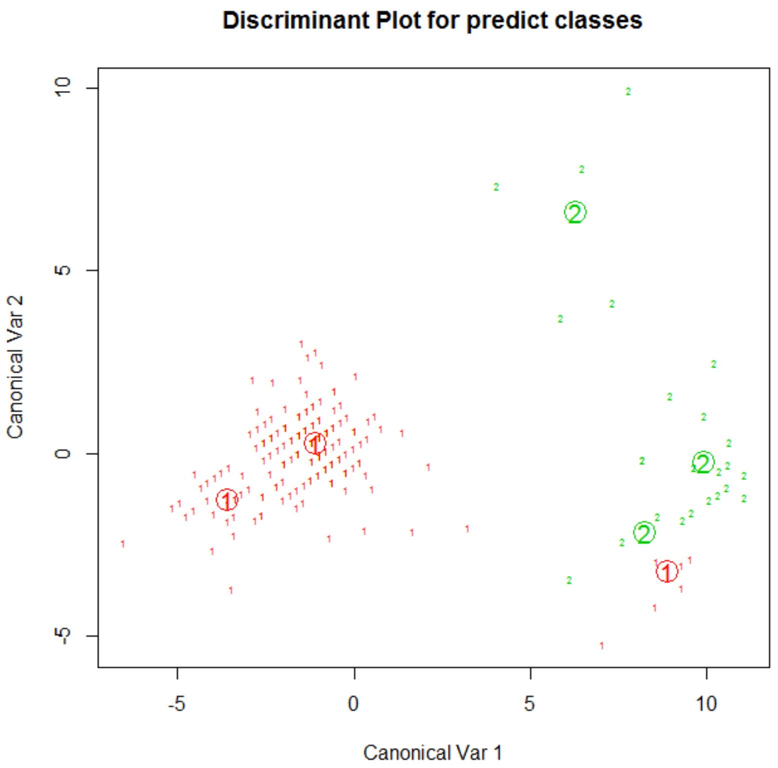

Figure S3. Two-dimensional view of the MDA model fitted to body size measurements. The points are independent sample data projected on to the leading two canonical coordinates. The subclass centers are indicated. Red figures: unparasitized *Azteca chartifex* larvae; green figures: parasitized larvae.

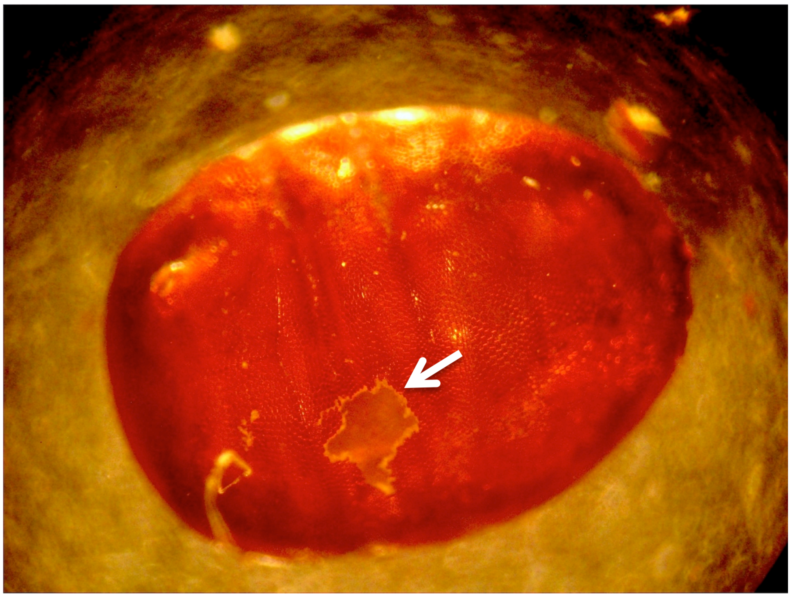

Figure S4. ***Nothomicrodon*** **puparium.** This individual has been parasitized; arrow points at the exit hole of the (unknown) parasitoid(s). Photo G. Pérez-Lachaud.

Table S1. List of included taxa and GenBank accession numbers for COIb sequences.

Family	Species	GenBank accession COIb
Lonchopteridae	*Lonchoptera tristis* Meigen	HM062534
Phoridae	*Apocephalus paraponerae* Borgmeier	AF217481
	Apodicrania molinai Borgmeier	GU559947
	Diplonevra florea (Fabricius)	HM352612
	Diplonevra setigera Malloch	HM352586
	Dohrniphora anterospinalis Borgmeier	HM352599
	Gymnophora spiracularis Borgmeier	GU559948
	Megaselia sp.	KM224503
	Melaloncha variabilis Brown	EU068515
Incertae sedis	*Nothomicrodon* sp.	LT592268
Pipunculidae	*Chalarus spurius* (Fallén)	HS547919
	Eudorylas fusculus (Zetterstedt)	LT797567
	Jassidophaga villosa (von Roser)	AY261685
	Nephrocerus lapponicus Zetterstedt	HF547906
Platypezidae	*Agathomyia sexmaculata* (von Roser)	KC699450
	Microsania pectipennis (Meigen)	KC699469
	Paraplatypeza bicincta (Szilády)	KC699447
	Platypeza hirticeps Verrall	KC699445
	Polyporivora picta (Meigen)	KM224507
Syrphidae	*Cheilosia pedemontana* Rondani	AY533336
	Eristalis tenax (Linnaeus)	EU431482
	Metadon captum (Speiser)	LT797565
	Metadon inermis (Loew)	LT797566
	Microdon pictipennis (Macquart)	HF547950
	Merodon albifrons Meigen	DQ386312
	Merodon luteihumerus Marcos García, Vujić & Mengual	LT797564
	Pseudomicrodon sp.	HF547949
	Volucella pellucens (Linnaeus)	AY261689
Sciadoceridae	*Sciadocera rufomaculata* White	GU559946

Table S2. ***Azteca chartifex* complementary material.** Collected in 2015, examined for gyne parasitism rate; +: presence; -: absence. As larvae were not dissected, developmental stages of *Nothomicrodon* present in the parasitized larvae could not be secured.

**Nest indent.**	**Gynes**	**Gyne pupae**	**Workers**	**Worker larvae**	**Gyne larvae**	**Parasitized gyne larvae**	**Gyne parasitism rate (%)**	**Corrected gyne parasitism rate (%)[a]**	***Nothomicrodon* L_3 wandering in the host nest**	
2015/1	+	+	+	+	87	32	36.8	40.2	5	
2015/2	-	-	-	+	4	3	75.0	75.0	0	
2015/3	-	+	+	+	662	42	6.3	6.3	0	
2015/4	+	+	+	+	48	33	68.8	70.6	2	1 puparium
2015/7	+	+	+	+	863	30	3.5	3.5	0	
2015/8	+	+	+	+	106	5	4.7	4.7	0	
Total					1770	145	8.2	8.6	7	1

[a] Corrected to take into account the free wandering *Nothomicrodon* larvae and the presence of a puparium.

Table S3. Phorid ectoparasitoids of social insects larvae.

Ectoparasitoid species	Host species	Host family	Observations	References
Aenigmatias brevifrons (Schmitz)[a]	*Formica rufibarbis* Fabricius	Formicidae	Exact host identity not fully elucidated	(11–16)
Aenigmatias lubbockii (Verrall)[b]	*Formica fusca* Linnaeus	Formicidae		(12–14, 16, 17)
	Formica picea Nylander	Formicidae		(14, 16)
Megaselia aletiae (Comstock)	*Polistes exclamans* Viereck	Vespidae		(18)
Megaselia scalaris (Loew)	*Polistes canadensis* (Linnaeus)	Vespidae		(19)
	Agelaia testacea (Fabricius)[c]	Vespidae		(20)
Megaselia sp. nr. *scalaris* (Loew)	*Mischocyttarus labiatus* (Fabricius)	Vespidae		(21)

[a]Referred to as *Platyphora lubbocki* and *P. dorni*. [b]Referred to as *A. blattoides* Meinert and *A. highlandicus* Schmitz. [c]Referred to as *Stelopolybia testacea*.

Table S4. Phorid endoparasitoids of social insects larvae.

Endoparasitoid species	Host species	Host family	Observations	References
Apodicrania termitophila (Borgmeier)	*Solenopsis invicta* Buren	Formicidae		(22, 23)
Borophaga incrassata (Meigen)	*Apis mellifera* Linnaeus	Apidae	On fully grown honeybee larvae	(24, 25)
Nothomicrodon sp.	*Azteca chartifex* Forel	Formicidae		This work
Unidentified phorid species	*Formica fusca* Linnaeus	Formicidae	6 semipupae each containing a large maggot	(26)
Unidentified phorid species	*Ectatomma ruidum* (Roger)	Formicidae	2 larvae each containing a fully grown dipteran larva	(27)
Unidentified phorid species	*Gnamptogenys tortuolosa* (F. Smith)	Formicidae	8/15 larvae containing parasitoid maggots	(28)
Unidentified species (presumably phorid)	*Dolichoderus doriae* Emery	Formicidae	47/500 larvae contained 1 or 2 dipterous larvae	(29)
Unidentified species (presumably phorid)	*Pachycondyla* sp.	Formicidae		(30)
Unidentified species (presumably phorid)	*Technomyrmex albipes* (F. Smith)	Formicidae	75/114 ant larvae contained 1 or 2 dipterous larvae	(29)

Table S5. ***Azteca chartifex*** **nests that contained** ***Nothomicrodon***. Collected in 2012 and 2015 at CEPEC-CEPLAC (Cocoa Research Center) field station, Ilhéus, state of Bahia, Brazil.

Nest identification	Collecting date	Geographical coordinates	Supporting tree	Height above ground (cm)	Nest length (cm)	Minimum nest width (cm)	Maximum nest width (cm)
Aztc 017	27/04/2012	14°45'52.0"S 39°14'10.1"W	*Psidium guajava*	350	25; 21[a]	32; 24[a]	36; 37[a]
Aztc 032	21/09/2012	14°46'00.0"S 39°13'28.0"W	*Theobroma cacao*	60	60	27	35
Aztc 033	11/10/2012	14°46'01.0"S 39°13'28.0"W	*T. cacao*	150	73	24	45
2015/1	25/10/2015	14°45'22.6"S 39°13'42.9"W	*T. cacao*	155	40	36	20
2015/2	26/10/2015	14°45'11.3"S 39°13'39.6"W	*T. cacao*	225	30	26	21
2015/3	26/10/2015	14°45'10.2"S 39°13'35.2"W	*T. cacao*	200	32	31	14
2015/4	28/10/2015	14°45'18.0"S 39°13'37.5"W	*T. cacao*	156	30	45	30
2015/7	31/10/2015	14°45'31.6"S 39°13'47.2"W	*T. cacao*	110	54	35	28
2015/8	01/11/2015	14°45'31.6"S 39°13'47.2"W	*T. cacao*	150	60	29	24

[a]Measures from two nests: a destroyed nest with few ants on the ground, and a new one constructed in its original place.
